# Neglected Microplastics and Their Risks in Rivers Throughout the Three Gorges Reservoir Area

**DOI:** 10.3390/toxics13090781

**Published:** 2025-09-15

**Authors:** Jian-Yun Xie, Bo Li, Qun-Po Jia, Xiao Li, Qin Zhao, Pei-Dang Fan, Chang-Qing Wang, Liu-Yi Zhang

**Affiliations:** 1 Three Gorges·Reservoir Area Environment·and·Ecology of Chongqing Observation and Research Station, Chongqing Key Laboratory of Water Environment Evolution and Pollution Prevention and Control in Three Gorges Reservoir Area, Chongqing Three Gorges University, Chongqing 404000, China; 15181897663@163.com (J.-Y.X.); 18388010424@163.com (Q.Z.); 18388822143@163.com (P.-D.F.); 17723271937@163.com (C.-Q.W.); 2 Eco-Environmental Monitoring and Scientific Research Center, Yellow River Basin Ecology and Environment Administration, Zhengzhou 450004, China; jia061096@163.com; 3 College of River and Ocean Engineering, Chongqing Jiaotong University, Chongqing 400074, China; naonao_li@163.com

**Keywords:** Three Gorges Reservoir, microplastic, occurrence characteristic, pollution risk, ecological risk

## Abstract

Microplastics have become a global environmental issue, and this challenge has also emerged in river environments. In this study, the Three Gorges Reservoir (TGR) was selected as the research area, and microplastics in water and sediments were chosen as the objects. The occurrence characteristics of microplastics were presented in rivers of the TGR through the methods of field sample collection and laboratory testing, and the risks of microplastics were evaluated using the pollution risk index (PRI) and potential ecological risk index. The results showed that the average abundances of microplastics in water and sediments of the rivers from the TGR were 15,464 particles/m^3^ and 1838 particles/kg, respectively. Furthermore, the main colors of microplastics in water and sediments of the TGR were black and blue, and the particle sizes were concentrated in the range of 200 to 500 μm. Polypropylene and polyethylene were the major polymers of microplastics in the water of the TGR, while the polypropylene–polyethylene copolymer was dominant in sediments. The PRIs in the water of the Yangtze River and its tributary from the TGR area were 2.45 and 1.72, respectively, and their PRIs were 2.43 and 2.01 in sediments, respectively, showing a low risk of pollution (level I). The average comprehensive potential ecological risk indices (RIs) for the Yangtze River and its tributaries were 27.28 and 18.82 in the water of the TGR, respectively, indicating low and moderate risk grades. However, there was a significantly high risk in sediment, and the average RI was 130.57 for the Yangtze River and 70.05 for the tributaries. Importantly, the neglected risks of microplastics in the river environment of the TGR area have been revealed, which provides a critical basis for the prevention and control of microplastic pollution here.

## 1. Introduction

Microplastics, recognized as an emerging type of environmental contaminants, have evolved into a global ecological concern, and their sources, environmental behaviors, occurrence patterns, and ecological risks are currently attracting significant attention [1,2]. With the continuous growth of plastic production and consumption globally, microplastic pollution is becoming increasingly prominent across aquatic environments, including rivers, lakes, reservoirs, and oceans [3]. A global annual input of plastic from rivers into the oceans ranges from 1.15 to 2.41 million tons, with a dominant contribution from rivers of the Asian continent [4]. The emerging microplastic pollution in rivers of the Three Gorges Reservoir (TGR) area, an integral component of the Yangtze River Basin, is particularly noteworthy. The abundances of microplastics in sediments and water have reached 43.95 ± 27.09 n/kg and 6214 ± 5394 particles/m^3^ here, respectively [5,6]. High-risk polymers of microplastics have been detected in sediments of several Yangtze tributaries, including polyethylene and polypropylene [7]. In contrast to other aquatic ecosystems, studies on microplastics in reservoirs accounted for only 2% of the global research effort [8], and most have primarily focused on localized areas or single media within these reservoirs. Consequently, the distribution patterns and risks of microplastics in water and sediments throughout the TGR area urgently require comprehensive investigation and assessment.

Global research on environmental microplastic behaviors has predominantly focused on marine systems, with less attention on freshwater systems [9]; however, microplastic pollution in these aquatic ecosystems represents a growing concern. Microplastic pollution reached moderate to high levels in the water and sediments of two major rivers in Harbin, China, posing substantial ecological risks [10]. Microplastic pollution in the river system is influenced by regional rainfall, topography, and other factors. For instance, pre-monsoon surface water exhibited higher microplastic abundance (1808 ± 697 particles/L) compared to post-monsoon surface water (1561 ± 167 particles/L) in the Mula River, and unregulated disposal of industrial waste is a potential source of microplastics [11]. The presence of microplastics in rivers has been documented across multiple regions, including Asia [12], Japan [13], and South Africa [14]. Additionally, there was a significant difference in microplastic abundances in the sediments from the South African reservoirs associated with human activity [15]. The microplastics in the reservoirs in the Shaying River Basin have also been investigated, and the transportation, suspension, and deposition of microplastics at different dam locations have been affected by dam construction, resulting in wide variations in abundance, color, shape, and size of microplastics in the water and sediments [16]. The ubiquitous microplastics pose intricate challenges to freshwater systems, such as rivers and reservoirs, underscoring the critical need for further research.

Microplastics pose multifaceted threats to aquatic ecosystems through organism ingestion, pollutant absorption and transport, habitat structure destruction, weakening of water self-purification capacity, and damage to biodiversity and genetic integrity, which ultimately degrade river ecosystem functionality and cause long-term imbalance [17,18]. Moreover, the risks of microplastics in river ecosystems are increasing due to their ability to adsorb organic matter, heavy metals, and other toxic substances [19]. Effective ecological risk assessment of riverine microplastics would serve as the guide for global plastic waste management strategies [20]. Microplastics have been identified significant ecological risk in sediments of the Karnaphuli River, particularly in areas with frequent agricultural and industrial activities [21]. Due to the inappropriate management of plastic waste in Khulna City, the Rupsha River is polluted by microplastics, harming both the mangrove and river organisms [22]. There are also large amounts of microplastics in the water of the Pearl River, China [23]. Obviously, the risks of microplastics have reached a worrying and dangerous level in river ecosystems, especially in areas with frequent human activities, and the ecological risk assessment and management of microplastics have become urgent.

The TGR area is a typical ecologically sensitive and vulnerable region, where microplastic behaviors and ecological effects in the aquatic ecosystem remain the research priority. It has been confirmed that microplastics are present in the water and sediments of rivers from the TGR, exhibiting notably higher abundances near the dam compared to other areas [24]. Moreover, microplastics in the water of the TGR mainly originated from anthropogenic activities in the watershed, and the extreme flood accelerated the transport of microplastics into the aquatic environment, resulting in a 57.9% increase in microplastics here [6]. The output of microplastics in the sediment of the TGR reached 8048 ± 7494 tons annually from 2008 to 2020, equivalent to 47 ± 44% of the microplastic productions poured from the Yangtze River into the sea [25]. Biomineralization processes preferentially settle lower-buoyancy microplastics in surface sediments, thereby amplifying ecological risks [26]. Most studies have been confined to specific river reaches or a single medium [27], making it difficult to comprehensively characterize the integrated distribution of microplastics across the complex aquatic environment of the TGR area. Generally, it has formed a vicious pattern for the microplastic generation, enrichment, transference, and sedimentation in the aquatic system due to the convergence of unique hydrological conditions and intensive human activities, positioning the TGR as a high-risk microplastic accumulation hotspot in the Yangtze River Basin.

Microplastics can distribute in different water layers when they enter the aquatic environment, affecting the aquatic ecosystem and posing a risk to human health via bioaccumulation in the food chain [28]. Therefore, the TGR was selected as the research area, and microplastics in river water and sediments were chosen as the research objects, aiming to (a) present the abundance of microplastics in water and sediments of the TGR through field sampling and laboratory analysis; (b) investigate their occurrence patterns, including their macroscopic and microscopic characteristics; and (c) assess their risks using the multi-method integration approach. In this study, comprehensive large-scale sampling was conducted in the water-sediment system of the key area throughout the TGR area, and the pollution risk index and potential ecological risk index were simultaneously applied to assess the risk of microplastics. The findings of this study provide a novel perspective for evaluating the risks of microplastics in aquatic environments.

## 2. Materials and Methods

### 2.1. Study Area

Since the Three Gorges Dam was completed in 2009, the TGR has become the world’s largest river-type reservoir [29]. The TGR is located in the eastern section of the upper reaches of the Yangtze River, stretching from Yichang City, Hubei Province, in the east to Jiangjin District, Chongqing City, in the west, China. The climate in the TGR area is subtropical monsoon, with an average annual precipitation of 1000 to 1800 mm. As a typical ecosystem with significant human intervention, the TGR area features a unique hydro-fluctuation belt and rich biodiversity. Meanwhile, it plays an important social and economic role, such as flood control, power generation, and shipping. However, its ecological uniqueness also brings environmental challenges. Multiple sources of microplastic pollution have emerged, and it may be attributed to the large reservoir area, dense urban and agricultural activities, and frequent vessel traffic, posing potential threats to the accumulation of microplastics in water and sediments.

### 2.2. Sample Collection

**Figure 1:**
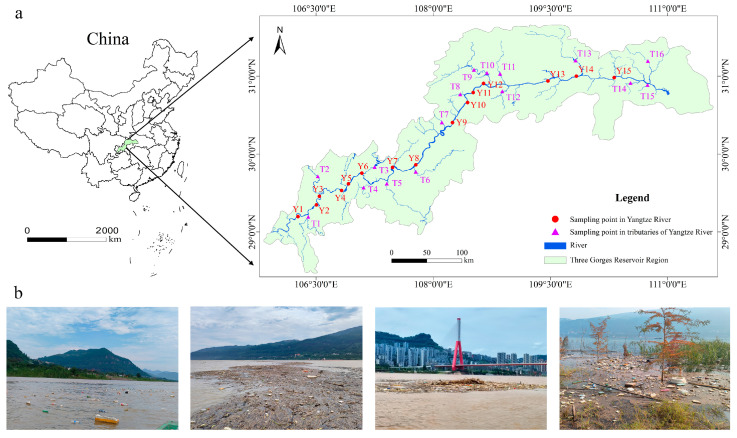
Sampling points of water and sediment in the TGR (**a**) and the plastics floating here (**b**).

The water and sediment samples were collected from the TGR in August 2024. A total of 31 sampling points were distributed in the TGR area (Figure 1). There were 15 sampling points in the Yangtze River of the TGR (Y1 to Y15), and 16 sampling points in the tributaries of the Yangtze River from the TGR (T1 to T16). These sampling points were relatively evenly distributed throughout the TGR Area. The feasibility of the sampling work and the distance between sampling points were the main factors in the design process. The surface sediments (0 to 10 cm) in the TGR were collected using a grab sampler (Changzhou Pun Sen Electronic Instrument Factory, Changzhou, China), and the surface water (5 L) was collected through a stainless steel water sampler (Changzhou Pun Sen Electronic Instrument Factory, Changzhou, China). The water was initially filtered using a 200-mesh and 400-mesh stainless steel screen in the field. Then, the retained substances on the sieves were rinsed into the glass bottle (1 L) with ultrapure water.

### 2.3. Extraction and Identification of Microplastics

The density separation flotation method was used to extract microplastics from the sediments. The 100 g sediments (dry weight) were added to the 1000 mL beaker, followed by the addition of 500 mL of ZnCl_2_ solution with a density of 1.5 to 1.6 g/cm^3^. The mixture was thoroughly stirred with a glass rod. Subsequently, the beaker was covered with the aluminum foil and left for 24 h, and the microplastics in the sediment would naturally float to the surface of the mixed solution. Then, the supernatant was filtered through the 200-mesh and 400-mesh stainless steel sieves, and the retained substances on the sieves were rinsed into the glass bottle (1 L) with ultrapure water. The above separation steps were repeated three times for the complete separation of microplastics from sediments. There may still be organic substances in the solution after the separation, which could disturb the subsequent identification of microplastics. Therefore, 200 to 300 mL of H_2_O_2_ solution was added for further elimination [30], and the solution was left for 24 h to remove organic matter. The treated solution was filtered through a vacuum filtration device (Gongyi Yuhua Instrument Co., Ltd., Zhengzhou, China), and then the microplastics were transferred to the glass fiber filter membrane (Zhengcheng Research Experiment Platform, Shanghai, China) with a pore size of 0.22 μm. It was placed in an oven (Shanghai Qixin Scientific Instrument Co., Ltd., Shanghai, China) at a temperature of 40 °C for drying. When the microplastics were extracted from the water, the H_2_O_2_ solution was added for the elimination of organic matter in the concentrated solution. The subsequent steps were the same as those for the sediment treatment described above.

A stereoscopic microscope (Nikon, SMZ800N, Tokyo, Japan) was used to identify the microplastics, including the color, shape, size, and abundance. The microplastic sizes were measured using Image View software 4.11. In order to further identify the type of microplastic polymer, the suspected microplastics were selected for detection through a micro-Fourier transform infrared spectroscopy (Thermo, Nicolet iS20, Waltham, MA, USA), and its wavelength identification range was set at 640 to 4000 nm.

### 2.4. Quality Assurance and Quality Control (QA/QC)

All solutions used for MPs collection were passed through the glass fiber filter membrane with a pore size of 0.22 μm. All glass implements used in the experiments were rinsed three times with ultrapure water. The whole process of the experiment was completed in a super clean room (Hunan Changhai Modern Laboratory Equipment Co., Ltd., Changsha, China), and wearing 100% cotton lab clothes. Furthermore, 221 and 357 particles suspected microplastics were detected in water and sediments, and the accuracy of microplastic selection was 98%. Three blank control groups were set to assess the potential environmental contamination during the experimental process, and they were processed for the same steps as the environmental samples. The blank value was subtracted from the sample measurements when the data analysis was carried out. To verify the accuracy of the extraction method for microplastics in sediments, the recovery experiment was conducted using the method by Li et al. [30]. Finally, the recovery rates of PP and PE were 97% and 96%, respectively.

### 2.5. Risk Assessment

The pollution risk index (PRI) that was proposed by Tomlinson et al. [31] was used to assess the microplastic pollution risks of river water and sediments in the TGR. The method was calculated as follows:

(1) $${\mathrm{PRI}}_{i}=\frac{C_{i}}{C_{0}}$$

(2) $${\mathrm{PRI}}_{\mathrm{TGR}}=\sqrt[{n}]{{\mathrm{PRI}}_{1}\times {\mathrm{PRI}}_{2}\times {\mathrm{PRI}}_{3}\times \ldots \ldots {\mathrm{PRI}}_{n}}$$

where *i* is the sampling point, and *n* is the number of sampling points. *C_i_* is the abundance of microplastics at the sampling point *i*, and *C*_0_ is the safe abundance of microplastics. The estimated safe abundance of microplastics in the surface water is 6650 particles/m^3^ [32]. The lowest detected microplastic abundance (770 particles/kg) in sediments of this study was considered as the safe concentration [33]. PRI_TGR_ is the pollution risk index of microplastics in the overall study area. In conformity with the calculated PRI, the microplastic pollution risk was categorized into four levels: <10 (level I), 10 to 20 (level II), 20 to 30 (level III), and >30 (level IV) [34].

The potential ecological risk index was utilized for further microplastic ecological risk to the ecosystem [33], and the method was calculated as follows:

(3) $$E_{r}^{i}=T_{r}^{i}\times \frac{C_{i}}{C_{n}^{i}}$$

(4) $$\mathrm{RI}=\sum_{i=1}^{m}E_{r}^{i}$$

where $E_{r}^{i}$ is the single risk index for microplastic polymer *i*, $T_{r}^{i}$ is the toxicity response coefficient of polymer *i*, *C_i_* is the detected polymer *i* abundance, and $C_{n}^{i}$ is the safe abundance of microplastic polymer *i*. Additionally, RI is the comprehensive potential ecological risk index for microplastics, and *m* is the number of microplastic polymers. The highest score of the microplastic polymer was selected for $T_{r}^{i}$, and the $T_{r}^{i}$ of the polypropylene–polyethylene copolymer (PP-PE), PP, and PE were 12, 1, and 11, respectively [35]. The improved ecological risk assessment criteria proposed by Li et al. [33] were used to evaluate the potential ecological risks of microplastics in water and sediment of the TGR (Table 1).

### 2.6. Statistical Analysis

The measurement unit for sediment is particles/kg, and the calculation unit for water is particles/m^3^. The results were expressed as mean value ± standard deviation. The significant difference in the data was compared through one-way analysis of variance or a non-parametric test using SPSS 19.0. The normal distribution and correlation analysis of data were also performed through SPSS 19.0. The experimental results were statistically analyzed using Excel 2016, and the graphs were plotted using Origin 9.0. ArcMap 10.2.2 was used to map the distributions of sampling points in the TGR.

## 3. Results and Discussions

### 3.1. Abundances of Microplastics in Water and Sediments

**Figure 2:**
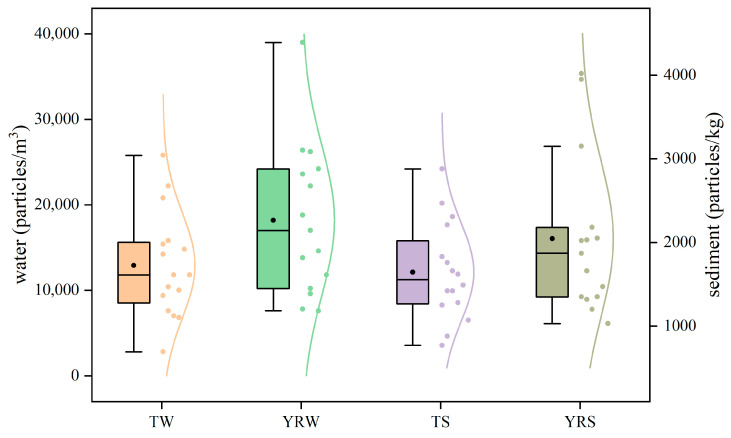
Abundances of microplastics in water and sediments of the TGR (TW: tributary water, YRW: Yangtze River water, TS: tributary sediment, YRS: Yangtze River sediment).

The microplastic abundance was 15,465 ± 7747 particles/m^3^ in the water of the TGR, and varied from 2800 to 39,000 particles/m^3^. They were 18,167 ± 8480 and 12,913 ± 5951 particles/m^3^ in the water of the Yangtze River and the tributaries here, respectively, and there was no significant difference (*r* = 0.06, *p* < 0.05). Meanwhile, the microplastic abundance was 1838 ± 780 particles/kg in the sediments of the TGR. They were 2044 ± 913 and 1645 ± 565 particles/kg in sediments of the Yangtze River and the tributaries, respectively, and there was also no significant difference (*r* = 0.17, *p* < 0.05). It was worth noting that the average microplastic abundances in water and sediments of the Yangtze River were higher than those in the tributaries from the TGR (Figure 2). There was a significant positive correlation of microplastic abundance in water and sediments from the TGR (*r* = 0.374, *p* < 0.05). The Yangtze River flows through densely populated areas, urban agglomerations, and industrial zones, where human activities directly lead to a large amount of microplastic inputs, such as garbage discharge, wastewater treatment plant effluent, agricultural runoff, etc. [36,37]. The tributary region of the TGR is predominantly rural or natural areas with relatively few pollution sources of microplastics. Importantly, the tributary system helps to collect microplastics into the Yangtze River [38] and leads to the Yangtze River becoming the sink for microplastics [33]. It was also found that the microplastic abundances in water and sediments from the region close to the urban area of Chongqing City were higher compared to other regions (for example, Y6 and T5 sampling points), and it further validated the results of Xu et al. [39].

The microplastic abundances of the river water in the TGR are higher than those of several freshwater systems worldwide (Table 2), and they cannot be neglected. It was similar to the report in Danjiangkou Reservoir in China [40], but significantly lower than the levels observed in the Liujiaxai Reservoir of the upper reaches of the Yellow River [24]. The elevated microplastic levels in the water of the TGR were attributed to the unique hydrological and anthropogenic characteristics [41]. Fortunately, the average microplastic abundance in sediments of the TGR reflected a lower level compared to global reservoir sediments [42], and it was similar to the previous conclusions [43]. The microplastic abundance in sediments of the TGR was extraordinarily serious in comparison with that from the Shuangtazi, Daliao Rivers, and the Liujiaxia Reservoir in China (Table 2), and the annual seasonal variations in water levels further exacerbate the sedimentation of microplastics [44]. The results in this study also show significant differences from other research conducted in the TGR [45], which highlighted methodological inconsistencies, such as the differences in sampling tools and analytical techniques. The investigation expanded the geographical coverage of sample collection and reduced the statistical errors caused by partial sampling.

### 3.2. Composition Characteristics of Microplastics

**Figure 3:**
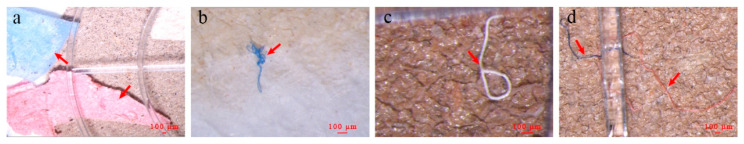
Representative microplastic specimens in water ((**a**): blue and red fragments, (**b**): blue line) and sediments (**c**): white line, (**d**): blue and red lines) from the TGR.

Microplastics were ubiquitously detected across all sampling sites in this study, exhibiting distinct colors, broad size spectrums, and diverse morphologies (Figure 3). The average proportion of microplastic colors had the characteristics of black > blue > red > green > transparent > yellow >white > purple > white in the Yangtze River water of the TGR, while it was black > blue > green > transparent > red > white > yellow > purple in the tributary water of the TGR. The black microplastics may originate from tire wear due to the presence of intensive road traffic in the TGR [53]. Furthermore, black microplastics were prone to photodegradation due to their strong light absorption property [54], and their rapid fragmentation led to the high abundance here.

**Figure 4:**
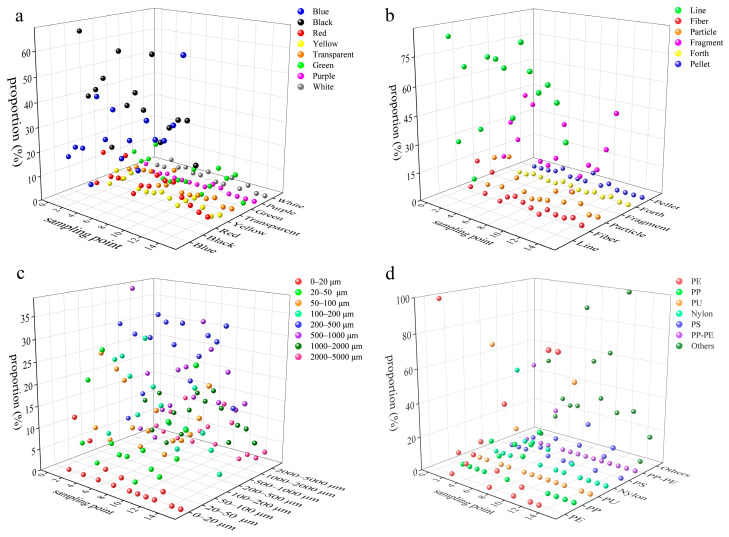
The microplastics colors (**a**), shapes (**b**), sizes (**c**), and polymer types (**d**) in the Yangtze River water of the TGR.

**Figure 5:**
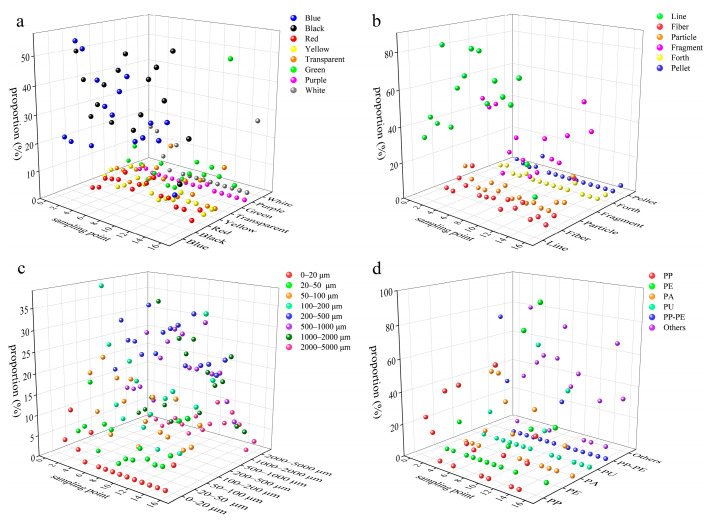
The microplastics colors (**a**), shapes (**b**), sizes (**c**), and polymer types (**d**) in the tributary water of the TGR.

The average proportion of line (exceeded 50%) and fragment (exceeded 20%) microplastics in the Yangtze River water of the TGR was higher than others (Figure 4), and it was similar to that in the tributary water (Figure 5); the results were consistent with the findings from other reservoirs globally [41]. Specifically, the high abundance of line microplastics in water is attributed to their low density, high buoyancy, and difficulty of disposal. The microplastic size was concentrated in the range of 200 to 500 μm in the Yangtze River and tributary water of the TGR, with average proportions of 27% and 26%, respectively. In contrast, the proportion of microplastics in the range of 0 to 20 μm was relatively low, with an average proportion of 2% here. The slow-flowing environment of the TGR promoted the retention of larger microplastics, while the small-sized microplastics were transferred to the deep-water layer through vertical migration [55]. Additionally, it is worth noting that the limitations of the detection methods may lead to an underestimation of small-sized microplastics, such as the pore size of the filter membrane.

**Figure 6:**
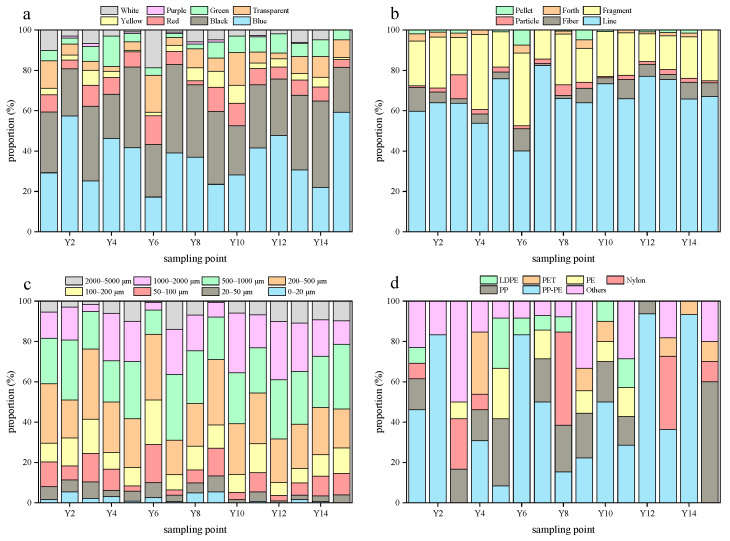
The characteristics of microplastics colors (**a**), shapes (**b**), sizes (**c**), and polymer types (**d**) in the Yangtze River sediments of the TGR.

**Figure 7:**
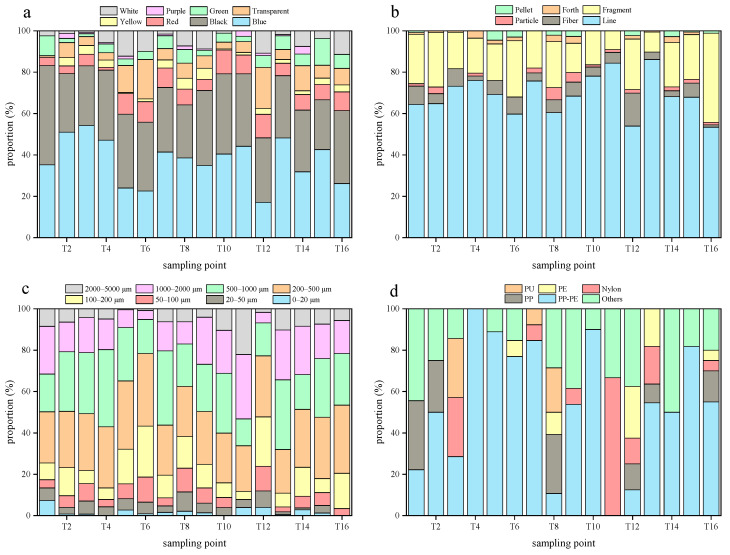
The characteristics of microplastics colors (**a**), shapes (**b**), sizes (**c**), and polymer types (**d**) in tributary sediments of the TGR.

Blue and black were the main colors of microplastics in the Yangtze River sediments of the TGR (Figure 6), with the average proportion of 36% and 32%, respectively. Meanwhile, they still accounted for a large proportion in sediments of the tributaries (Figure 7), and their average proportions were 37% and 33%, respectively. There results were akin to the findings from the sediments below the Three Gorges Dam [5]. The surface color of dark microplastics changes minimally during the photolysis process, and they are more easily identified visually compared to transparent or light-colored microplastics [5]. Notably, the color characteristics of microplastics in sediments from the Yangtze River and the tributaries in the TGR were prominently similar, and it was speculated that this similarity arises from either the mixing of microplastics throughout the reservoir via hydraulic transportation or the presence of common pollution sources, including shipping and agricultural runoff [56].

Line microplastics accounted for the largest proportion (66%) in the Yangtze River sediments of the TGR, followed by fragment microplastics. The average proportion of line microplastics was 69% in the tributary sediments, and 21% for fragment microplastics. Pellet microplastics only accounted for a small proportion in the TGR sediments, with an average proportion of 1% in the Yangtze River and tributaries. They were more readily suspended because of the low density, and it was ratiocinated that a minor part of them would enter the sediment through biological sedimentation or absorption of organic matter. These distribution characteristics confirmed the dominant role of the hydraulic sorting effect in the occurrence of microplastics in sediments, which was consistent with the general pattern of microplastics in sediments from reservoirs [57]. The microplastic sizes ranging from 200 to 500 μm have the highest average proportion (25%) in the Yangtze River sediments of the TGR. Moreover, the microplastic size in tributary sediments was mainly concentrated in the range of 200 to 500 μm (27%). The density separation method used in this study failed to identify microplastics of small size due to the limitations of the extraction and identification methods of microplastics in sediments [58], and the ZnCl_2_ used also posed a potential contamination risk during this process. Accordingly, the techniques of extracting and identifying microplastics in sediments need to be further optimized, and the focal plane array-based micro-Fourier transform infrared technique can be considered [59].

The various microplastic polymers were found in water and sediments of the TGR (Table 3). PE and PP microplastics were the predominant polymer types in the water of the TGR. Nevertheless, the sediments in the Yangtze River and its tributaries from the TGR were dominated by PP-PE microplastics, with the proportions being 45% and 54%, respectively. The occurrence of these polymers can be attributed to various plastic products. For instance, PP is extensively applied in items like disposable plastic bags, food containers, garbage liners, and wrapping films [60]. The microplastics from personal care products typically include PE and PP, which are used to improve product consistency [60]. PP and PE are the two types of plastics with the largest global production, and they are widely used in packaging films, daily necessities, agricultural mulching films, etc. [61]. The dense urban and rural activities and agricultural cultivation around the TGR have exacerbated the use and disposal of such materials. The suspension migration ability of PP-PE microplastics was extensive due to their density being close to that of the water, while the density increased after being bio-attached or adsorbed with particulate matter, and they sank into the sediments ultimately. PP and PE monomers or copolymers were difficult to degrade in natural environments [62], which was one of the reasons why these microplastics were stored in large quantities in sediments here.

### 3.3. Risks of Microplastics

**Figure 8:**
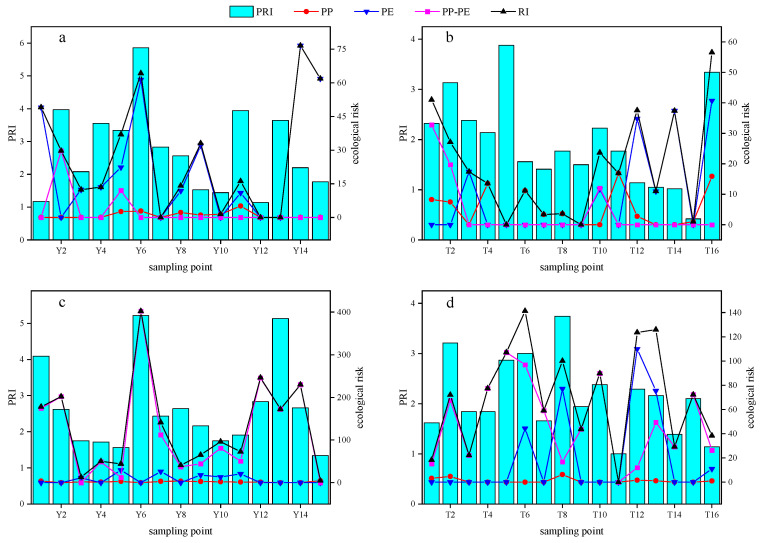
The risk assessments of microplastics in water and sediments of the TGR ((**a**): Yangtze River water, (**b**): tributary water, (**c**): Yangtze River sediment, (**d**): tributary sediment).

The PRIs were 2.45 ± 1.28 and 1.71 ± 0.89 in the Yangtze River and tributary water of the TGR, respectively, both of which belong to the pollution risk of level I. It was also found that the PRI in the Yangtze River sediments was greater than that in the tributaries of the TGR (Figure 8). The pollution risk posed by microplastics in water and sediments of the TGR is primarily determined by their abundance [63], and the high pollution risk of microplastics was observed in the Yangtze River water (sampling point Y6). It stemmed from the local intensive industrial activity and its position near the confluence of the Yangtze River and Longxi River, and the decreased flow velocity promoted microplastic accumulation here. The high pollution risk of microplastics may be elevated due to less effective waste management practices in the confluence of the Yangtze River and its tributaries of the TGR [64]. Consequently, it is essential to explore the transport mechanisms of microplastics in Jialing (sampling point T2), Wu (sampling point T5), Zhuxi (sampling point T8), and Xiangxi Rivers (sampling point T16), where the pollution risk of microplastics was serious, which is crucial for the development of effective strategies for microplastic management throughout the TGR area.

The single ecological risk indices of PP, PE, and PP-PE microplastics in the Yangtze River water of the TGR were 1.01 ± 1.50, 23.48 ± 25.47, and 2.78 ± 7.80, respectively. It was worth mentioning that the PE microplastics in the Yangtze River water have reached extremely high-risk ecological grades at the sampling points Y1, Y6, Y14, and Y15, which were located in the Jiangjin, Changshou, and Wushan Districts of Chongqing City, and Enshi City of Hubei Province, respectively. The PP, PE, and PP-PE microplastics were all at low ecological risk grades in the tributary water. Furthermore, the Yangtze River sediments of the TGR showed a low ecological risk of PP and PE microplastics, whereas an extremely high ecological risk of PP-PE was found here. The single ecological risk index of PP, PE, and PP-PE microplastics in tributary sediments of the TGR were 1.12 ± 1.87, 19.87 ± 34.92, and 49.06 ± 32.35, which were low, moderate, and extremely ecological grades, respectively.

The RI of microplastics in the Yangtze River water of the TGR was 27.28 ± 24.79, and approximately 20% of the surveyed areas were classified as posing a severe ecological risk. Significantly, the ecological risks associated with microplastics were found to be more severe in sediments than in the water of the TGR. The RI for microplastics in the Yangtze River sediments of the TGR reached 130.57 ± 105.45, and there were extremely high ecological risk levels for 53.33% of sampling points. The RI of microplastics in tributary sediments was 70.05 ± 41.22, and 37.5% of sampling points reached an extremely high ecological risk level. On the one hand, the high levels of heavy metals and persistent organic pollutants were attracted to the microplastics in the aquatic environment of the TGR [65,66], and the ecological risks caused by co-contaminant adsorption require further attention. Moreover, the microplastic ingestion rates by local biota are not yet known, and the RI is an overall ecological indicator. On the other hand, the microplastics that have been retained by the aquatic plants have increased the ecological risks in the TGR through phytostabilization [67], but this needs to be confirmed in future investigations. Notably, the PP, PE, and PP-PE microplastics have been identified as the predominant polymer types in the water and sediments of the TGR, and they pose a relatively high ecological risk. Their sources need to be strictly controlled to reduce the ecological risks, and measures to reduce plastic waste and improve the recycling of agricultural plastic films should be considered.

## 4. Conclusions

The average abundance of microplastics in water and sediments of the TGR was 15,464 particles/m^3^ and 1838 particles/kg, and it was higher in the Yangtze River than in the tributaries here. The microplastics in the water and sediments of the TGR were predominantly black and blue, and the sizes were primarily distributed in the range of 200 to 500 μm. The PP and PE were the dominant microplastic polymer types in the water of the TGR, whereas the PP-PE copolymer prevailed in sediments. The PRIs in the water of the Yangtze River and its tributary from the TGR were 2.45 and 1.72, and they were 2.43 and 2.01 in sediments, respectively, which were all at a level I pollution risk. From the perspective of the RI of microplastics, the average RIs for the Yangtze River and its tributaries were 27.28 and 18.82 in water of the TGR, reaching 130.57 and 70.05 in sediments of the Yangtze River and tributaries from the TGR, respectively. Microplastic pollution in the TGR arises from the superposition of natural processes and human activities. Therefore, the complete chain control system of source reduction, process interruption, and terminus treatment can be implemented. Here, the neglected microplastics and their risks in rivers throughout the TGR area have been revealed, providing a critical basis for the precise prevention and control of microplastic pollution in this area. Unfortunately, this study was unable to reflect the dynamic changes of microplastics because only one season of data was available.

## Figures and Tables

**Table 1:** Grading standard of potential ecological risks of microplastics and polymers in water and sediments from the TGR.

Grade	I	II	III	IV	V
$E_{r}^{i}$	$E_{r}^{i}$ < 10	10 ≤ $E_{r}^{i}$ < 20	20 ≤ $E_{r}^{i}$ < 30	30 ≤ $E_{r}^{i}$ < 40	$E_{r}^{i}$ ≥ 40
RI	RI < 20	20 ≤ RI < 40	40 ≤ RI < 60	60 ≤ RI < 80	RI ≥ 80
Risk level	low	moderate	high	severe	extremely

**Table 2:** Comparison of microplastic abundances in water and sediments around the world.

	Area	Unit	Abundance	Mean Abundance	Reference
Water	TGR	particles/m^3^	2800~39,000	15,465	This study
	TGR	items/m^3^	/	2614 ± 297	[41]
	TGR	items/m^3^	/	4895 ± 3670	[43]
	TGR	particles/m^3^	/	6214 ± 5394	[25]
	Danjiangkou Reservoir (China)	particles/m^3^	530~24,798	7205	[40]
	Liujiaxia Reservoir (China)	particles/m^3^	4.48 × 10^6^~12.09 × 10^6^	/	[24]
	Babura River (Indonesia)	particles/m^3^	6.8 × 10^4^~13.2 × 10^4^	/	[46]
	River Ganga (India)	particles/m^3^	51 ± 7	26 ± 4	[47]
Sediments	TGR	particles/kg	770~4020	1838	This study
	TGR	items/kg	/	286 ± 229	[43]
	Shuangtaizi River (China)	n/kg	67~300	170 ± 96	[48]
	Daliao River (China)	n/kg	100~476	237 ± 129	[48]
	Yan River (China)	n/kg	208~686.67	444.95	[49]
	Danjiangkou Reservoir (China)	Items/kg	/	3989	[50]
	Liujiaxia Reservoir (China)	Item/kg	447.27~1543.80	/	[24]
	Brishbane River (Australia)	Items/kg	10~520	/	[51]
	Citanduy River (Indonesia)	n/kg	18,190~70,405	/	[52]

/ There is no data.

**Table 3:** The microplastic polymer types in water and sediments of the TGR (TW: tributary water, YRW: Yangtze River water, TS: tributary sediment, YRS: Yangtze River sediment).

Microplastic Polymer Type	Abbreviation	YRW (%)	TW (%)	YRS (%)	TS (%)
Polyethylene	PE	19.68	17.02	5.20	4.89
Polypropylene	PP	12.60	18.09	16.18	10.33
Polyurethane	PU	10.24	9.57	1.73	4.89
Nylon	Nylon	9.45	4.26	9.25	5.44
Polystyrene	PS	7.87	2.13	4.05	1.09
Polypropylene–polyethylene copolymer	PP-PE	6.30	7.45	44.51	53.8
Polyamide	PA	5.51	12.76	1.16	1.63
Polyethylene terephthalate	PET	3.93	1.06	5.20	1.09
Phenol resin	PR	3.15	5.32	/	/
polybutadiene	PB	1.57	2.13	1.16	/
Polyolefin	PO	0.79	1.06	1.16	1.63
Polyester	PES	0.79	1.06	/	/
Low-density polyethylene	LDPE	0.79	/	5.20	2.17
Polymethacrylate–polystyrene copolymer	PAM-PS	0.79	/	1.73	2.17
Others	Others	16.54	18.09	3.47	10.87

/ There is no data.

## Data Availability

Data is contained within the article.
